# Downregulation of YAP-dependent Nupr1 promotes tumor-repopulating cell growth in soft matrices

**DOI:** 10.1038/oncsis.2016.29

**Published:** 2016-04-18

**Authors:** Q Jia, W Zhou, W Yao, F Yang, S Zhang, R Singh, J Chen, J J Chen, Y Zhang, F Wei, Y Zhang, H Jia, N Wang

**Affiliations:** 1 Laboratory for Cellular Biomechanics and Regenerative Medicine, Department of Biomechanical Engineering, School of Life Sciences and Technology Huazhong University of Science and Technology, Wuhan, Hubei, China; 2 Department of Mechanical Science and Engineering, College of Engineering, University of Illinois at Urbana-Champaign, Urbana, IL, USA

## Abstract

Despite decades of significant progress in understanding the molecular mechanisms of malignant tumorigenic cells, it remains elusive what these tumorigenic cells are and what controls the growth of these malignant cells. Recently, we have mechanically selected and grown highly malignant and tumorigenic tumor-repopulating cells (TRCs), a small sub-population of cancer cells, by culturing single cancer cells in soft fibrin matrices. However, it is unclear what regulates TRC growth besides Sox2. Here we show that nuclear protein 1 (Nupr1), a protein independent of Sox2, is downregulated in TRCs of melanoma, ovarian cancer and breast cancer cultured in soft fibrin matrices. *Nupr1* expression depends on nuclear translocation of YAP that is enriched at the *Nupr1* promoter sites; YAP is controlled by Cdc42-mediated F-actin and Lats1 interactions. Nupr1 regulates tumor-suppressor p53 and negatively regulates Nestin and Tert that are independent of Sox2 and promote TRC growth. Silencing *Nupr1* increases TRC growth and Nupr1 overexpression inhibits TRC growth in culture and in immune-competent mice. Our results suggest that Nupr1 is a suppressor of growth of highly tumorigenic TRCs and may have a critical role in cancer progression.

## Introduction

Despite decades of continuous efforts and significant progress in understanding the molecular mechanisms of malignant tumorigenic cells,^1^ it remains unresolved what these tumorigenic cells are and what controls the growth and proliferation of these tumor cells. Recently, we mechanically selected tumorigenic tumor-repopulating cells (TRCs) independent of surface stem cell markers, a small sub-population of cancer cells, by culturing single cancer cells from cancer cell lines or primary tumors in three-dimensional (3D) soft fibrin matrices.^2^ These TRCs exhibit highly tumorigenic and proliferative capacity and they efficiently repopulate tumors at distant organs in wild-type syngeneic and non-syngeneic mice.^2^ The soft fibrin matrices promote TRC growth by facilitating Sox2 expression.^3^ However, the melanoma cells can significantly increase their proliferation when Sox2 expression is only modestly increased,^3^ suggesting that other factors that are regulated by matrix softness may contribute to TRC proliferation independent of Sox2. Using microarrays to screen for potential factors, we have identified nuclear protein 1 (Nupr1) that is markedly reduced in the TRCs in 3D soft fibrin matrices when compared with melanoma cells on 2D rigid plastic. Nupr1 is a DNA-binding protein implicated in regulation of cell cycle and apoptosis.^4^ Nupr1 is also called COM1 (candidate of metastasis 1) because it is expressed in metastatic cancer cells.^5^ The exact role of Nupr1 in cancer is poorly understood as several studies have associated Nupr1 with cancer cells' ability to survive^6, 7, 8, 9^ while another shows its tumor-suppressing capabilities.^10^ Here we reveal that Nupr1 is downregulated in TRCs to facilitate proliferation of the tumorigenic cells and its expression depends on nuclear Yes-associated protein (YAP)/transcriptional coactivator with PDZ-binding motif (TAZ).

## Results

### Substrate stiffness regulates Sox2-independent Nupr1

**Figure 1:**
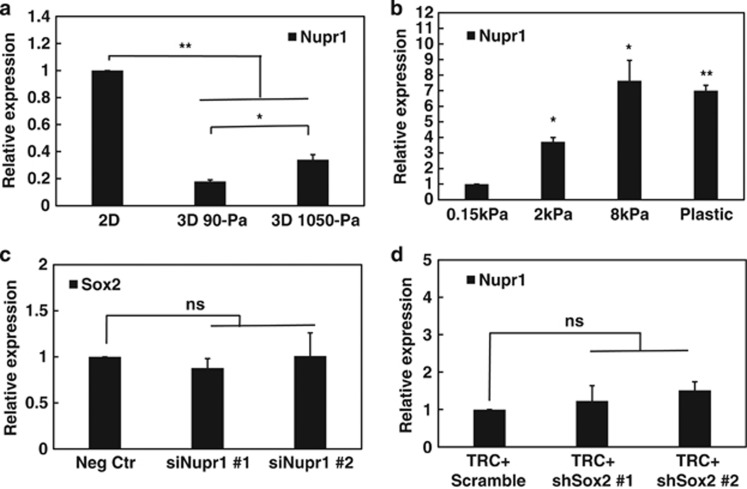
*Nupr1* is low in soft matrix and independent of Sox2. (**a**) *Nupr1* mRNA expression is lower in 3D fibrin gels than on rigid plastic. 2D: B16-F1 cells were cultured on rigid plastic. 3D 90-Pa, 3D 1050-Pa: cells were cultured in 90 or 1050-Pa fibrin gels for 5 days. After 5 days, the mRNA was extracted for quantitative analysis of *Nupr1* mRNA expression by real-time PCR. Cells that are grown in 3D 90 Pa gels are defined as TRCs. (**b**) *Nupr1* mRNA expression increases with substrate stiffness. Cells were cultured on the surface of collagen-1-coated polyacrylamide gels of various stiffnesses or on glass-bottomed dishes for 24 h. All were compared with ‘0.15 kPa'. (**c**) Silencing *Nupr1* does not affect *Sox2* expression. Cells were transfected with negative control siRNA, or *Nupr1* siRNA #1, #2, for 24 h respectively, and then analyzed for *Sox2* mRNA expression by real-time PCR. (**d**) Silencing *Sox2* do not affect *Nupr1* expression. TRCs were extracted from 3D fibrin gels and re-plated onto 2D 90-Pa fibrin gel surface for 3 h, and then transfected with Scramble control, or *Sox2* shRNA#1, #2, for 12 h (or for 24 h, Supplementary Figure 10c) and then analyzed for *Nupr1* mRNA expression by real-time PCR. Mean±s.e.m.; *n*=3 independent experiments for all subfigures; *P<0.05; **P<0.01; NS, not statistically significant.

To determine the impact of matrix rigidity on *Nupr1*, we compared its expression in cells that are cultured on 2D rigid plastic with that in cells in 3D soft fibrin matrices. *Nupr1* mRNA expression was decreased by ~70% in 3D soft (90-Pa) fibrin matrices (Figure 1a) and Nupr1 protein levels were decreased considerably (Supplementary Figure 1a) when compared with that on 2D rigid plastic. *Nupr1* expression was also much lower in human ovarian cancer cells (A2780) and human breast cancer cells (MCF-7) cultured in 3D soft fibrin gels (Supplementary Figure 1b), suggesting that low mRNA expression of *Nupr1* is not limited to melanoma cells in 3D soft substrates. Increasing 3D matrix rigidity from 90 to 1050-Pa significantly upregulated *Nupr1* expression (Figure 1a). We define the melanoma cells that are selected and grown in 90-Pa fibrin gels for 5 days as TRCs.^3^ Importantly, *Nupr1* expression in the cells plated on 2D substrates increased by >7-fold when substrate stiffness was increased from 150-Pa to 8 kPa or rigid plastic (Figure 1b), suggesting that it is the substrate rigidity and not the substrate dimensionality that dictates *Nupr1* gene expression. Silencing *Nupr1* had no effect on *Sox2* expression (Figure 1c); silencing *Sox2* had no effect on *Nupr1* expression either (Figure 1d). These data suggest that *Nupr1* can be regulated by matrix rigidity and that *Nupr1* is independent of *Sox2*.

### Little YAP translocation to TRC nucleus

**Figure 2:**
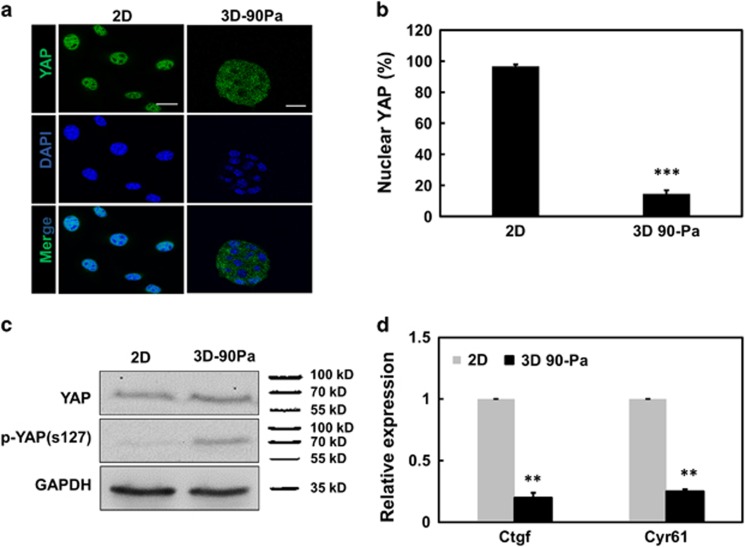
Nuclear YAP is low in 3D soft matrix. (**a**) Confocal immunofluorescence images of YAP and nuclei (4,6-diamidino-2-phenylindole (DAPI)) of cells on 2D rigid plastic (left column) or in 3D 90-Pa (right column) fibrin gel. Cells were culture on 2D rigid dishes or in 90-Pa 3D fibrin gels for 3 days. Scar bars, 20 μm. (**b**) Percentage of cells with predominantly nuclear YAP. Mean±s.e.m.; *n*=10 randomly chosen view-fields; ***P<0.001 (**c**) Western blotting for phosphorylated YAP S127 (p-YAP s127) and YAP in whole-cell lysates of melanoma cells after 3 days of culture on 2D rigid plastic or in 3D 90-Pa fibrin gels. (**d**) Real-time PCR of *Ctgf* and *Cyr61* as indexes of YAP transcriptional activity on 2D and in 90-Pa 3D fibrin gels. Mean±s.e.m.; *n*=3; **P<0.01.

As Nupr1, a nuclear protein, responds to substrate rigidity, we wondered what may be its upstream regulator(s). YAP, and its transcriptional coactivator TAZ, are known as mediators of Hippo signaling and organ growth,^11, 12^ shuttling between the cytoplasm and the nucleus, where they interact with transcriptional enhancer associate domain transcription factors to regulate transcription. YAP is known to sense cytoskeletal tension and mediate cellular mechanoresponses.^13^ We assayed endogenous YAP subcellular localization by immunofluorescence for cells plated on 2D rigid plastic and in 3D 90-Pa substrates. Almost all YAP in cells on 2D rigid plastic was nuclear, whereas only ~18% YAP in cells in 3D 90-Pa fibrin matrices was nuclear (Figures 2a and b). Furthermore, immunoblots show that 3D 90-Pa fibrin gels promoted YAP phosphorylation on serine 127 (ser^127^), whereas the total amounts of YAP in 2D and in 3D 90-Pa were similar (Figure 2c), consistent with published results that phosphorylated YAP do not translocate to the nucleus.^14, 15^ *Ctgf* and *Cyr61*, two known YAP/TAZ endogenous targets, were used as markers of transcriptional activity of YAP. *Ctgf* and *Cyr61* mRNA expression levels in cells in 3D 90-Pa were markedly reduced when compared with those on 2D (Figure 2d), supporting the finding that little YAP from TRCs (the cells in 3D 90-Pa) was translocated from the cytoplasm to the nucleus.

### Cdc42 regulates Nupr1 via YAP

**Figure 3:**
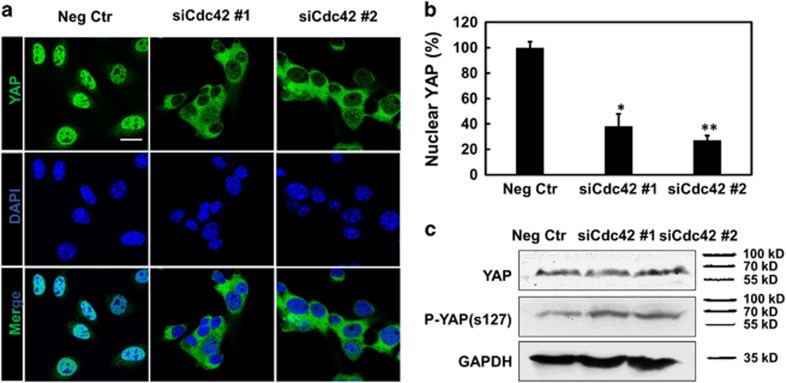
Silencing *Cdc42* downregulates nuclear YAP. (**a**) Confocal immunofluorescence images of YAP and nuclei (4,6-diamidino-2-phenylindole (DAPI)). Cells were treated with negative control siRNA, or *Cdc42* siRNA #1, #2 for 24 h, respectively. Scar bar, 20 μm. (**b**) Percentage of cells with predominantly nuclear YAP. Mean±s.e.m; *n*=10 randomly chosen view-fields. *P<0.05; **P<0.01. (**c**) Western blotting for phosphorylated YAP S127 (p-YAP s127) and YAP in whole-cell lysates of melanoma cells. Control cells were transfected with negative control siRNA, or *Cdc42* siRNA #1, #2 for 24 h, respectively.

**Figure 4:**
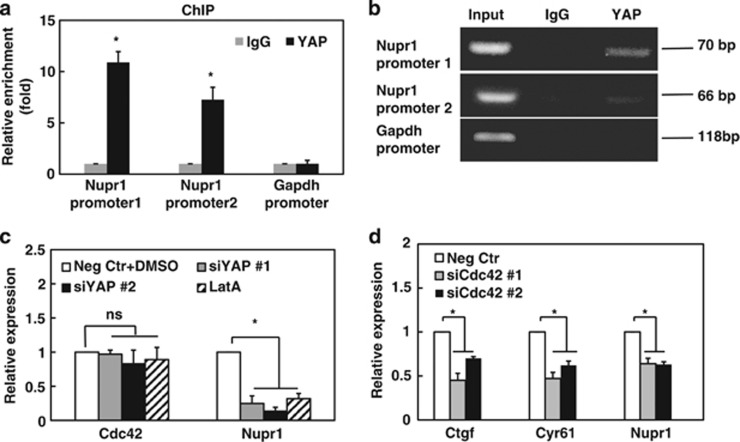
Silencing *Cdc42* downregulates nuclear YAP enriched at Nupr1 promoter sites. (**a**) YAP is enriched at Nupr1 promoter sites, assayed using ChIP assay. ChIP was performed using normal rabbit IgG (negative control) or YAP antibody on control cells lysates. Two sets of primers were used for *Nupr1* promoter region. To exclude nonspecific effect, the relative enrichment of Gapdh promoter was also shown. Relative enrichment was determined by real-time PCR. Mean±s.e.m.; *n*=3; *P<0.05. (**b**) Representative blots of the reverse transcriptase–PCR (RT–PCR). Similar results are observed in two other blots. (**c**) YAP impacts *Nupr1* expression. Control cells were treated with negative control siRNA, DMSO, YAP siRNA#1 or #2 for 24 h, or 1 μM Latrunculin A (LatA), and then analyzed for *Nupr1* mRNA expression by real-time PCR. Silencing YAP with siRNA#1 or #2 markedly reduced YAP protein levels (Supplementary Figure 11a). (**d**) Silencing *Cdc42* decreases expression of *Ctgf, Cyr61* and *Nupr1*. Cells were transfected with negative control siRNA, or *Cdc42* siRNA#1, #2 for 24 h, respectively, and then analyzed for Ctgf, Cyr61 and *Nupr1* mRNA expression by real-time PCR. Mean±s.e.m.; *n*=3 independent experiments for all subfigures; *P<0.05; NS, not statistically significant.

As a key cytoskeleton regulator, Cdc42 is known to regulate the actin cytoskeleton to promote filopodia formation.^16, 17^ We have reported that Cdc42 is significantly downregulated in TRCs compared with control melanoma cells (the cells on 2D rigid plastic).^3^ To investigate the relationship between Cdc42 and YAP, we knocked down *Cdc42* in the cells on 2D rigid plastic. In comparison with the cells transfected with negative control small interfering RNA (siRNA), the cells transfected with *Cdc42* siRNA exhibited dominant YAP cytoplasmic localization (Figures 3a and b) and high levels of phosphorylation of YAP on serine 127 (Figure 3c). To determine the role of nuclear YAP in *Nupr1*, we performed chromatin immunoprecipitation (ChIP) assay and found that YAP was enriched by 7- to 10-folds at Nupr1 promoter sites for melanoma cells on 2D rigid plastic (Figures 4a and b). For cells cultured in 3D 90-Pa fibrin gels, the YAP enrichment was reduced by ~70% when compared with those on 2D rigid plastic (Supplementary Figure 2a), suggesting the specific role of YAP in *Nupr1* gene expression. Silencing *YAP* downregulated *Nupr1* expression but had no effect in *Cdc42* expression (Figure 4c). Knocking down transcriptional enhancer associate domains (TEADs) markedly reduced YAP enrichments for cells on 2D rigid plastic (Supplementary Figure 2a) and inhibited *Nupr1* expression (Supplementary Figure 2b), suggesting that YAP requires the presence of transcriptional enhancer associate domains to regulate *Nupr1* expression at *Nupr1* promoter sites. Furthermore, silencing *Taz* in cells on rigid plastic downregulated Nupr1 (Supplementary Figure 3a), which was significantly lower in TRCs than in cells on rigid plastic (Supplementary Figure 3b). Disrupting F-actin with latrunculin A (Supplementary Figures 4a and c) elevated phosphorylated YAP (Supplementary Figure 5a), increased cytoplasmic YAP (Supplementary Figure 5b) and decreased nuclear YAP (Supplementary Figure 5c). Latrunculin A had no effect on *Cdc42* expression but decreased *Nupr1* expression (Figure 4c) consistent with published reports that F-actin interacts with *Lats1*, which in turn regulates phosphorylation of YAP.^14^ Phosphorylated-YAP was higher in TRCs than in cells on rigid plastic and silencing *Cdc42* elevated phosphorylated YAP (Supplementary Figure 6). In addition, silencing *Cdc42* downregulated YAP activation markers *Ctgf* and *Cyr61,* and also *Nupr1* (Figure 4d), likely via lowering F-actin (Supplementary Figures 4b and d) to free up *Lats1* so that it can phosphorylate YAP. This conclusion was supported by the results that knocking down *Lats1/2* increased nuclear translocation of YAP by twofolds (from ~20 to ~60%) (Supplementary Figures 7a and b) and upregulated *Nupr1* gene expression (Supplementary Figure 7c). Together, these data suggest that Cdc42 that promotes assembly of F-actin and Lats1 binding to F-actin is upstream of YAP and regulates *Nupr1* via YAP translocation.

### Nupr1 is a negative regulator of melanoma cell growth *in vitro* and *in vivo*

**Figure 5:**
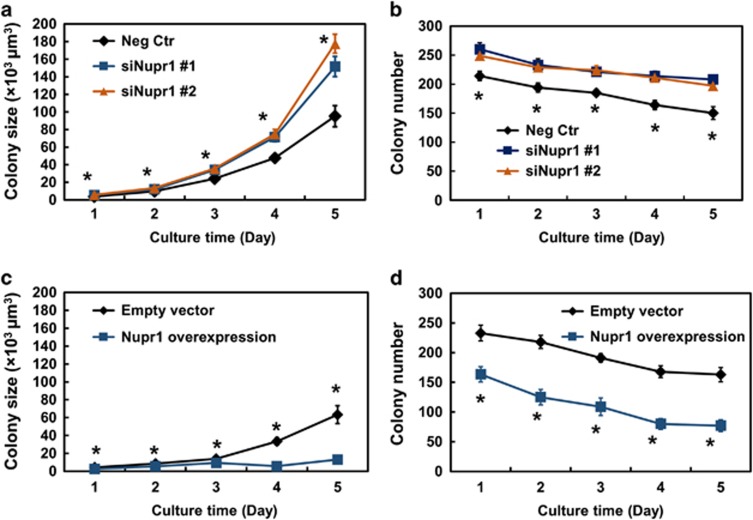
*Nupr1* negatively regulates colony growth in culture. Silencing *Nupr1* using two different siRNAs significantly increases colony size (**a**) and number (**b**). Cells were transfected with *Nupr1* siRNA #1 or #2 for 24 h, and then cultured in 90-Pa fibrin gels. Colony number and colony size were quantified until day 5. Mean±s.e.m.; *n*=2 independent experiments; *P<0.05, indicating significant differences between Neg Ctr and siNupr1 #1 or #2. (**c**, **d**) Overexpressing *Nupr1* for 24 h before plating significantly inhibits colony size and number. Significant differences between empty vector and Nupr1 overexpression from day 1 through day 5. Mean±s.e.m.; *n*=2 independent experiments, *P<0.05.

To determine the functional role of *Nupr1* in melanoma cells, we altered *Nupr1* levels and assayed melanoma cell proliferation. Compared with the cells grown in 3D 90-Pa gels transfected with negative controls, silencing *Nupr1* significantly increased colony size and colony number (Figures 5a and b; Supplementary Figures 8 and 9). In contrast, overexpressing *Nupr1* markedly reduced colony size and number (Figures 5c and d; Supplementary Figures 8 and 9). These results suggest that *Nupr1* negatively regulates TRC growth.

**Figure 6:**
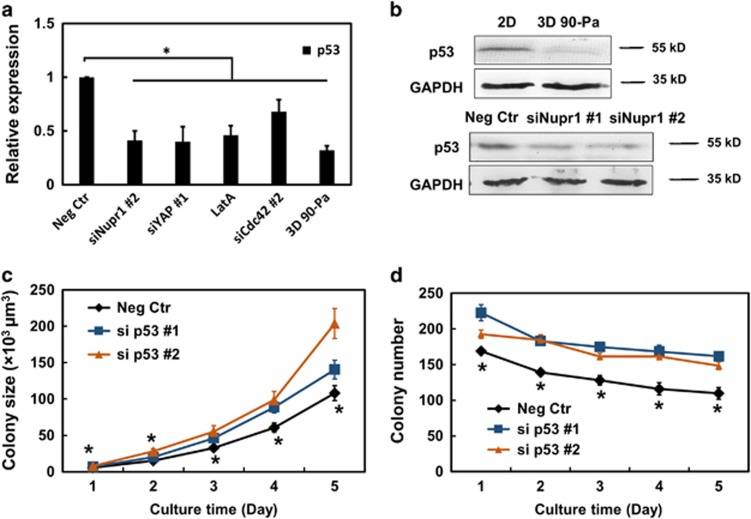
Nupr1 is upstream of tumor-suppressor p53. (**a**) p53 is lower in TRCs and silencing *Nupr1* decreases *p53* expression. Cells were treated with negative control siRNA, *Nupr1* siRNA#2, YAP siRNA#1, *Cdc42* siRNA#2 for 24 h, and 1 μM Latrunculin A for 12 h, or plated in 90-Pa fibrin gels, and then analyzed for *p53* mRNA expression by real-time PCR. Mean±s.e.m.; *n*=3 independent experiments. *P<0.05. (**b**) Western blotting assays for p53. 2D: cells were cultured on rigid plastic; 3D 90 Pa: cells cultured in 90-Pa fibrin gels for 5 days. Neg Ctr, siNupr1#1, siNupr1#2: cells were transfected with negative control siRNA, *Nupr1* siRNA#1, or #2 for 24 h. (**c**, **d**) Silencing p53 using two different siRNAs significantly increases colony size and number. Control cells were transfected with p53 siRNA #1 or #2 for 24 h, and then cultured in 90-pa fibrin gels. Colony number and size were quantified until day 5. Mean±s.e.m.; *n*=2 independent experiments; *P<0.05, between Neg Ctr and si p53 #1 or #2.

It is reported^8^ that Nupr1 interacts with tumor-suppressor p53. *p53* mRNA levels (Figure 6a) and protein levels (Figure 6b) were much lower in cells plated in 3D soft fibrin gels (TRCs) than in melanoma cells plated on 2D rigid plastic. Silencing *Nupr1* decreased both *p53* mRNA and protein levels considerably (Figures 6a and b); silencing *YAP* and *Cdc42* in cells on rigid plastic that are upstream of *Nupr1* or treating cells on rigid plastic with Latrunculin A to disrupt F-actin significantly decreased *p53* expression (Figure 6a). Together, these results suggest that *Nupr1* is upstream of p53. Silencing *p53* in cells on rigid plastic significantly increased colony size and colony number, consistently with the known role of p53 as a tumor suppressor (Figures 6c and d).

**Figure 7:**
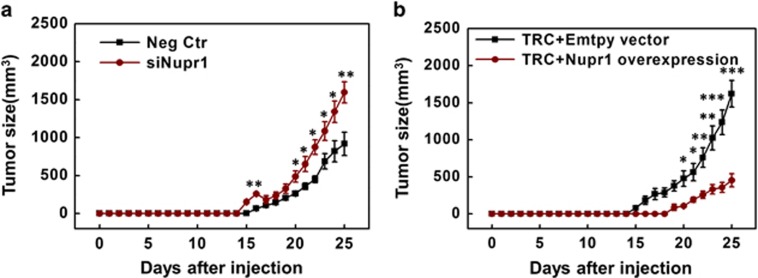
Nupr1 negatively regulates tumor growth *in vivo*. From the published results on limiting dilution assays, it is known that 100 subcutaneously injected TRCs but not 100 melanoma cells grown on 2D rigid plastic can generate tumors; when 100 000 cells grown on rigid plastic are injected, however, 100% mice have tumors,^2^ presumably a small population of the cells grown on rigid plastic are TRC-like cells.^2^ Hence, we chose 2000 tumor cells to inject subcutaneously in order to determine the potential differences in tumorigenic efficiency (see Table 1) and tumor growth rates when Nupr1 was perturbed. (**a**) In all, 2000 cells cultured on 2D rigid plastic were transfected with negative control siRNA (Neg Ctr) or Nupr1 siRNAs (siNupr1) for 24 h and then subcutaneously injected to mice. The tumor volume was quantified using the formula of length times the square of width times 0.52. (**b**) Overexpressing Nupr1 suppresses tumor growth. 2000 TRCs transfected with empty vector (TRC+empty vector) or Nupr1 complementary DNA plasmids (TRC+Nupr1 overexpression) for 12 h were subcutaneously injected to mice. The volume of skin melanoma was measured for 25 days. Mean±s.e.m.; *n*=9 (Neg Ctr) and 12 (siNupr1) in **a**; *n*=15 (empty vector) and 9 (Nupr1 overexpression) in **b**; data were pooled from two independent experiments as results from the two experiments were similar, in each experiment eight mice were used per condition; *P<0.05; **P<0.01; ***P<0.001. Welch's unpaired *t*-test.

To further explore the role of Nupr1 *in vivo*, we either silenced *Nupr1* in melanoma cells grown on 2D rigid plastic (whose Nupr1 levels were high; Figure 1a; Supplementary Figure 1a) or overexpressed *Nupr1* in TRCs (whose Nupr1 levels were low; Figure 1a; Supplementary Figure 1a) and injected those treated cells into mice at 2000 cells per mouse subcutaneously. Silencing *Nupr1* in 2D melanoma cells elevated the number of mice with tumors (from 9 out of 16 mice to 12 out of 16 mice); overexpressing *Nupr1* in TRCs significantly suppressed the number of mice with tumors (from 15 out of 16 mice to 9 out of 16 mice) (Table 1). Importantly, sizes of the tumors were significantly increased when *Nupr1* was silenced and were significantly suppressed when *Nupr1* was overexpressed (Figures 7a and b). These findings are consistent with the *in vitro* colony size results and support the notion that *Nupr1* behaves like a tumor-suppressor *in vivo*.

### Nupr1 negatively regulates nestin and Tert

**Figure 8:**
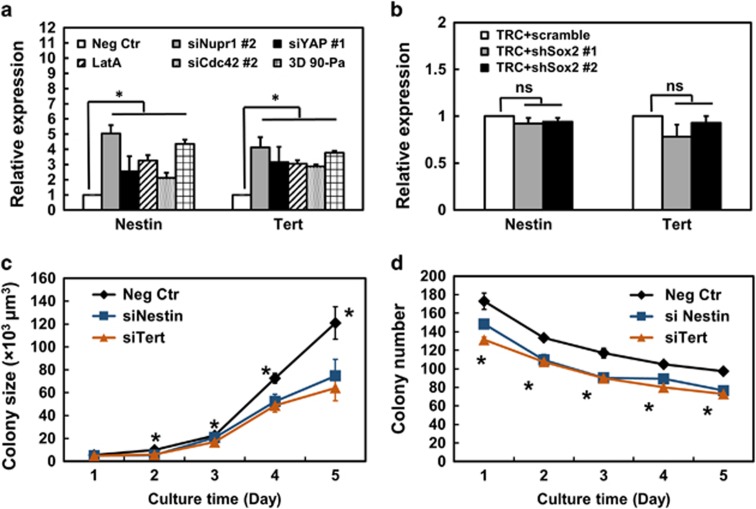
*Nestin* and *Tert* are independent of *Sox2* and promote cell proliferation. (**a**) Nestin and Tert are regulated via the Cdc42-YAP-Nupr1 pathway. Cells were treated with negative control siRNA, *Nupr1* siRNA#2, YAP siRNA#1, *Cdc42* siRNA #2 for 24 h, and 1 μM Latrunculin A for 12 h, or plated in 90-Pa fibrin gels, and then analyzed for *Nestin* and *Tert* mRNA expression by real-time PCR. Mean±s.e.m.; *n*=3 independent experiments. *P<0.05. (**b**) Silencing *Sox2* does not affect expression of *Nestin* and *Tert*. TRCs were transfected with scrambled control shRNA or *Sox2* shRNAs for 12 h and their mRNAs were used to quantify the expression levels of *Nestin* and *Tert* by real-time PCR. Mean±s.e.m.; *n*=3 independent experiments; NS, no statistical significance. Extending the shRNA *Sox2* transfection duration to 24 h did not have any effect on *Nestin* and *Tert* expression either (Supplementary Figure 10c). (**c**, **d**) Silencing *Nestin* or *Tert* significantly inhibits colony size and number. Cells were transfected with negative control, *Nestin* or *Tert* siRNA, and then cultured in 90-Pa fibrin gels. Colony number and colony size were quantified till day 5. Mean±s.e.m.; *n*=3 independent experiments; all *P*<0.05 between Neg Ctr and siNestin or siTert except at day 1 in **c**.

To further explore what else may be regulated by *Nupr1*, we examined *Nestin* and *Tert*, which are upregulated in TRCs^2^ (Figure 8a). Silencing *Sox2* had no effects on either Nestin or Tert, suggesting these two molecules are independent of *Sox2* (Figure 8b). Knocking down *Nupr1,* YAP or *Cdc42*, or disrupting F-actin with Latrunculin A, led to upregulation of *Nestin* and *Tert* (Supplementary Figure 8a), suggesting that *Nupr1* and molecules that are upstream of *Nupr1* negatively regulate *Nestin* and *Tert*. Importantly, silencing either *Nestin* or *Tert* decreased both colony size and colony number (Figures 8c and d), suggesting that *Nestin* and *Tert* facilitate cell growth. Together, these results suggest that *Nestin* and *Tert* are independent of *Sox2* and dependent on *Nupr1* and promote cell proliferation.

## Discussion

Our current reveals an important role of Nupr1 in inhibiting growth of tumorigenic cancer cells *in vitro* and *in vivo* and the pathway that is critical for Nupr1 expression. Published reports show that Sox2 is critical in pluripotency maintenance^18^ of embryonic stem cells and fate determination^19, 20^ of adult stem cells. Sox2 upregulation has been found in tumor-initiating cells or precursors of osteosarcomas,^21^ glioblastoma,^22^ lung and esophageal squamous cell carcinomas,^23^ and breast cancer.^24^ In our previous studies, we have shown that Sox2 is markedly upregulated in melanoma TRCs.^2, 3^ More recently, we have discovered an essential role of Sox2 in mediating efficient extravasation of melanoma TRCs via downregulating Cdc42 and thus F-actin in a zebrafish model.^25^ However, Sox2 may not be the only critical factor in various carcinomas as in many carcinomas there is no evidence that Sox2 is upregulated. Our current study reveals that Nupr1, independent of Sox2, is low in three types of carcinoma TRCs (melanoma, ovarian cancer and breast cancer) and is a negative regulator of TRC growth. We find that regulation of growth of TRCs by the soft mechanical microenvironment is complex. As the matrix stiffness becomes very low (~100 Pa), *Cdc42* is lowered, which leads to decreases in F-actin, which in turn frees up *Lats1* so that it binds to and phosphorylate YAP, preventing translocation of YAP into the nucleus. As a result, *Nupr1* is downregulated; downregulation of *Nupr1*, in turn, leads to downregulation of *p53* and upregulation of *Nestin* and *Ter*t. All these help to promote growth and self-renewal of TRCs (Supplementary Figure 10). This pathway appears to be independent of the Sox2-mediated pathway but is regulated by the soft matrices of the microenvironment. At the present, we do not know how important Nupr1 is in TRCs in many other carcinomas, but the abrogation of the inhibitory role of Nupr1 in soft matrices and the upregulation of Nupr1 in stiff matrices support the postulate of physical microenvironment barrier in cancer progression^26^ and are consistent with the role of 3D stiff matrices in inhibiting carcinoma progression.^3^ In the future, we need to determine how Nupr1, together with other tumor suppressors, inhibits carcinoma progression in human subjects and to find ways to perturb this pathway to stop cancer progression.

## Materials and methods

### Animals

Four-week old C57BL/6 female mice were obtained from Center of Medical Experimental Animals of Hubei Province (Wuhan, China). The mice were randomly assigned to be in the control group or the treated group. As a minimum of six mice per group was required for having a statistical power, each group had eight mice, The experimentalists were blinded from the expected outcome of the treatment. All animals received humane care in compliance with the Principles of Laboratory Animal Care Formulated by the National Society of Medical Research and the guide for the US National Institutes of Health. The protocol was approved by the Animal Care and Use Committee of Huazhong University of Science and Technology.

### Cell lines and cell culture

Human ovarian cancer cell line A2780, human MCF-7 breast cancer cell line and murine melanoma cell line B16-F1 were purchased from China Center for Type Culture Collection (CCTCC, Wuhan, China). Cells were cultured on rigid dishes with RPMI-1640, Dulbecco's modified Eagle's medium or minimum essential medium cell culture medium supplemented with 10% fetal bovine serum (Life Technologies, Carlsbad, CA, USA), and 1% penicillin and streptomycin at 37 °C with 5% CO_2_. Cells were passaged every 3–4 days using TrypLE (Life Technologies). Cell samples were randomly allocated to each well of the culture dishes and randomly chosen for intervention. For all cell culture experiments, at least three independent experiments were performed per condition.

### 3D fibrin gel preparation

Salmon fibrinogen and thrombin were purchased from Reagent Proteins (San Diego, CA, USA). 3D fibrin gels were prepared as described previously.^2, 3^ In brief, fibrinogen was diluted into 20 mg/ml with T7 buffer (pH 7.4, 50 mM Tris, 150 mM NaCl). Cells were detached from 2D rigid dishes. Fibrinogen and single-cell solution mixture was made by mixing the same volume of fibrinogen solution and cell solution, resulting in 1 or 8 mg/ml fibrin gels (the stiffness of 1 and 8 mg/ml fibrin gels is 90 and 1050 Pa, respectively). In all, 250 μl cell/fibrinogen mixture was seeded into each well of 24-well plate and mixed well with pre-added 5 μl thrombin (100 U/ml). The cell culture plate was then incubated in 37 °C cell culture incubator for 25 min. Finally, 1 ml of minimum essential medium containing 10% fetal bovine serum and antibiotics was added.

### 2D polyacrylamide gel preparation

Polyacrylamide gels were prepared using the protocol reported previously.^27^ Gel stiffness was varied by altering the concentrations of bis-acrylamide crosslinker (0.04%, 0.0.5% and 0.3%) and acrylamide (3%, 5% and 5%) and the corresponding gel stiffness is 0.15, 2 and 8.0 kPa, respectively. The gel surface was activated with Sulpho-SANPAH under ultraviolet light for 5 min and coated with 100 μg/ml collagen-1 overnight.

### Real-time qPCR analysis

Total mRNA was isolated from the cells using the Trizol reagent according to the supplier's instruction (Life Technologies). Reverse transcription (RT) was performed using the TransScript First-strand cDNA Synthesis Super Mix (TransGen, Beijing, China), according to the manufacturer's protocol. Real-time quantitative PCR (qPCR) was performed using GoTaq qPCR Master Mix (Promega, Madison, MI, USA). The data were normalized against mouse glyceraldehyde 3-phosphate dehydrogenase (GAPDH). The sequences of all the primers for real-time qPCR are listed in Supplementary Table 1.

### Immunofluorescence

B16 in plastic dish or 3D fibrin gel culture were fixed with the fix buffer consisting of 4% formaldehyde (BioLegend, Shanghai, China) for 10 min at room temperature. Cells were then permeabilized with 0.5% Triton X-100 (BioLegend) for 2 min and treated with blocking serum (Solarbio, Beijing, China, SL1) for >5 h. Primary antibody, anti-YAP (CST, Danvers, MA, USA, 1:100, #14074) for immunofluorescence was incubated overnight. After being washed to remove unbound primary antibodies, cells were incubated with the secondary antibody Alexa Fluor 488 (Abcam, Cambridge, MA, USA, 1:2000, ab150069) and 4,6-diamidino-2-phenylindole (Biosharp, Wuhan, China, BS130A) for 2 h under dark conditions. Images were acquired with a Leica SP2 confocal microscope (Mennheim, Germany). For quantifications of YAP subcellular localizations, YAP immunofluorescence signal was scored as predominantly nuclear versus evenly distributed/predominantly cytoplasmic in 80–100 cells for each experimental condition.

### Western blotting assay

To quantify the expression levels of YAP, phospho-YAP, Nupr1, cells were lysed with RIPA Lysis buffer (Beyotime, Jiangsu, China). Each sample were separated by 8–15% sodium dodecyl sulfate–polyacrylamide gel electrophoresis, blocked with 5% bovine serum albumin overnight at 4 °C and incubated with primary antibodies to YAP (rabbit, 1:1000, CST, #14074), phospho-YAP (Ser127) (rabbit, 1:1000, CST, #13008), p8 (T-14) (goat, 1:1000, Santa Cruz, Dallas, TX, USA, sc-23283) and GAPDH (mouse, 1:1000, Abcam, ab8245) for 2 h at room temperature. Primary antibodies were detected with goat anti-rabbit IgG-horseradish peroxidase (1:2000, Santa Cruz, sc-2004), donkey anti-goat IgG-horseradish peroxidase (1:2000, Santa Cruz, sc-2020) or anti-mouse IgG-horseradish peroxidase (1:2000, Santa Cruz, sc-2005). The blots were developed using Immobilon Western Chemiluminescent HRP Substrate (Millipore, Billerica, MA, USA).

### F-actin staining

Cells were fixed with 4% paraformaldehyde for 10 min at room temperature. The F-actin was stained using 0.76 mM rhodamine-phalloidin (Sigma, St Louis, MO, USA, 94072 Atto 565 phalloidin) and the nuclei was stained with 10 mg/ml 4,6-diamidino-2-phenylindole (Biosharp, BS130A) for 30 min at 37 °C. The samples were rinsed three times with 1X phosphate-buffered saline before imaging. F-actin content was quantified along the lines shown using Image J (NIH, Bethesda, MA, USA).

### ChIP assay

We performed ChIP assay following the manufacturer's instructions (EZ-ChIP kit, Millipore). Briefly, cells were subjected to cross-linking with 1% formaldehyde in medium for 10 min at 37 °C and then lysed on ice. Chromatin was sonicated to shear DNA to an average length of 0.2–1.0 kb. ChIP was performed using control rabbit IgG or antibody against YAP (CST, #14074). The immunoprecipitation was heated to reverse the formaldehyde cross-linking and the DNA fragments in the precipitates was purified for real-time qPCR and PCR analysis. Primers were sets corresponding to Nupr1 and GAPDH (negative control) promoter regions. The sequences of these promoter regions can be found in Transcriptional Regulatory Element Database (Cold Spring Harbor Laboratory, Long Island, NY, USA). Primers used were:

Mus Gapdh promoter: forward, 5'-TCTTCTTGTGCAGTGCCAGGT-3'; reverse, 5'-CACACTTCGCACCAGCATCC-3'. Mus Nupr1 promoter 1: forward, 5'-ACAAGGAGACACAGGCAAGACT-3'; reverse, 5'-TGGCTGTTGGTGGCAAGGT-3'.

Mus Nupr1 promoter 2: forward, 5'-TGCTTGGGTGAGTCCTGTGAG-3' reverse, 5'-GACAGCAGTCCTGAGCAGAGA-3'.

### Transfection

Cells were transfected with siRNA, short hairpin RNA (shRNA) or complementary DNA using Lipofectamine 2000 (Invitrogen) following the manufacturer's protocol. Silencer negative control no. 1 siRNA (Invitrogen, AM4611) was used a negative control in RNA interference experiment. The sequences of siRNA or shRNA are listed in Supplementary Table 2. The Nupr1 complementary was obtained from OriGene (Rockville, MD, USA, MG200140). pEGFP-N1 vector (Clotech, Mountain View, CA, USA) was used as control. Knocking down or overexpressing efficiency for various genes were shown in Supplementary Figure 11.

### Colony number assay

By changing the focal planes along the *z* axis (the direction of gel depth), the colony number was counted view by view. At least three wells of colonies were counted per condition per day.

### Statistical analysis

All statistics were performed using a two-tailed Student's *t*-test with unequal variance except the Fisher's exact test and Welch's unpaired *t*-test for analyzing data from mice experiments.

## Figures and Tables

**Table 1:** Nupr1 suppresses tumor growth *in vivo*

*Mouse model cell line*	*C57BL/6 mice (subcutaneous injection)*
*B16-F1 (2000 cells)*	
Neg Ctr (2D)	9/16
siNupr1 (2D)	12/16
TRC+empty vector	15/16
TRC+Nupr1 overexpression	9/16*

Abbreviations: 2D, two dimensional; Nupr1, nuclear protein 1; TRC, tumor-repopulating cell.

*P<0.05, Fisher's exact test.
