# Supplementary material for: Downregulation of YAP-dependent Nupr1 promotes tumor-repopulating cell growth in soft matrices
Source: Oncogenesis. 2016 Apr 18;5(4):e220–. doi: 10.1038/oncsis.2016.29 (PMC4848840; doi:10.1038/oncsis.2016.29)

**Supplementary Information**

**Downregulation of YAP-dependent Nupr1 promotes tumor-repopulating cell growth in soft matrices**

Qiong Jia^1^, Wenwen Zhou^1^, Wenting Yao^1^, Fang Yang^1^, Shuang Zhang^1^, Rishi Singh^2^, Junwei Chen^1^, Junjian Chen^1^, Yao Zhang^1^, Fuxiang Wei^1^, Yuejin Zhang^1^, Haibo Jia^1^，and Ning Wang^1, 2*^

^1^Laboratory for Cellular Biomechanics and Regenerative Medicine, Department of Biomechanical Engineering, School of Life Sciences, Huazhong University of Science and Technology, Wuhan, Hubei 430074 China

^2^Department of Mechanical Science and Engineering, College of Engineering, University of Illinois at Urbana-Champaign, Urbana, IL 61801 USA

Key words: tumor suppressor, tumor growth, matrix rigidity, 3D substrate

*Send correspondence to:

Dr. Ning Wang, [nwangrw@illinois.edu](mailto:nwangrw@illinois.edu)

**
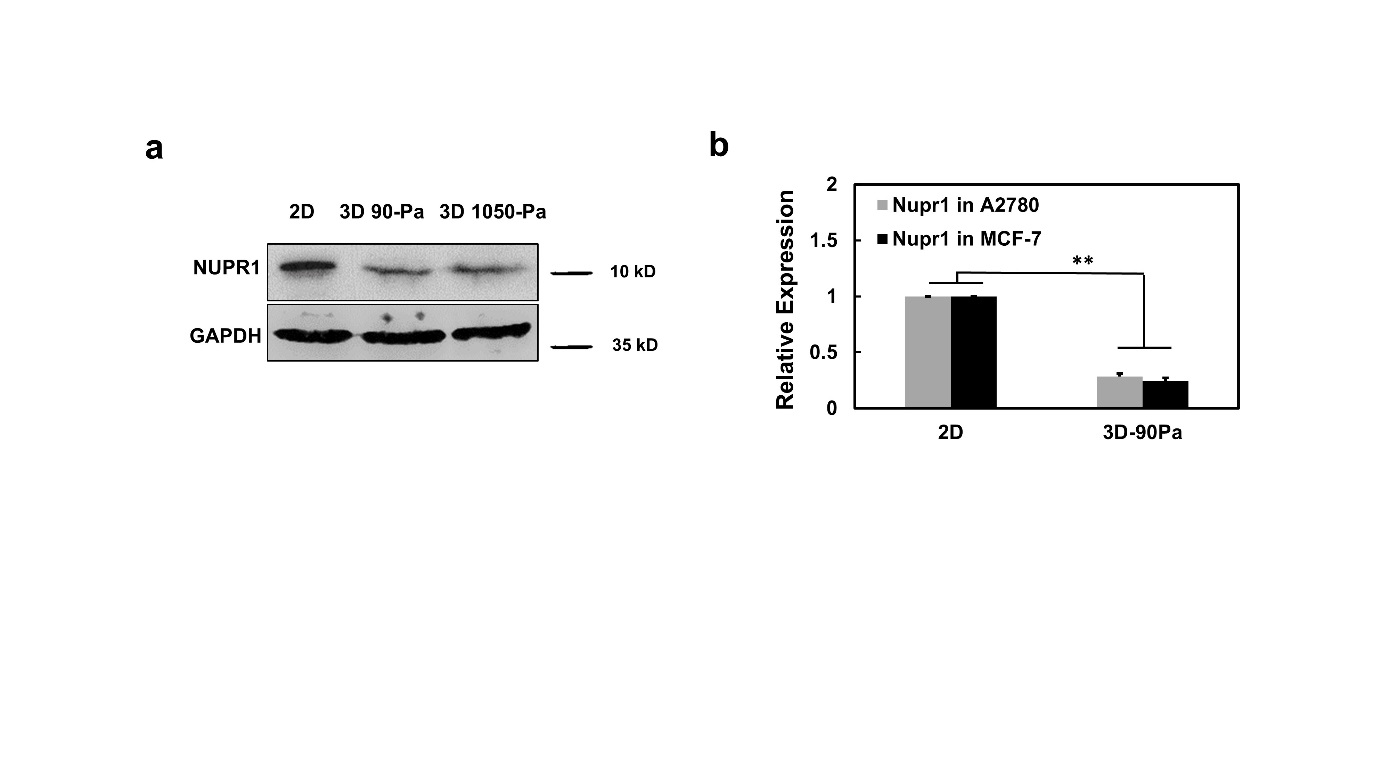
Supplementary Figure 1. Nupr1 decreases in TRCs. (a)** *Nupr1* protein level is lower in 3D fibrin gels than on rigid plastic. Cells were cultured on 2D rigid plastic or in 90-Pa or in 1050-Pa 3D fibrin gels, respectively. After 5 days, the protein was extracted for western blot analysis. Similar results were obtained in two other experiments. **(b)** *Nupr1* decreases in human ovarian cancer A2780 TRCs and human breast cancer MCF-7 TRCs. A2780 cells were cultured on rigid plastic (**2D**) or in 90-Pa fibrin gels (**3D-90Pa**) for 5 days. Total mRNAs were extracted to quantify the expression of *Nupr1* by real time PCR. Mean ± s.e.m.; n=3 independent experiments. ***p*<0.01.

**
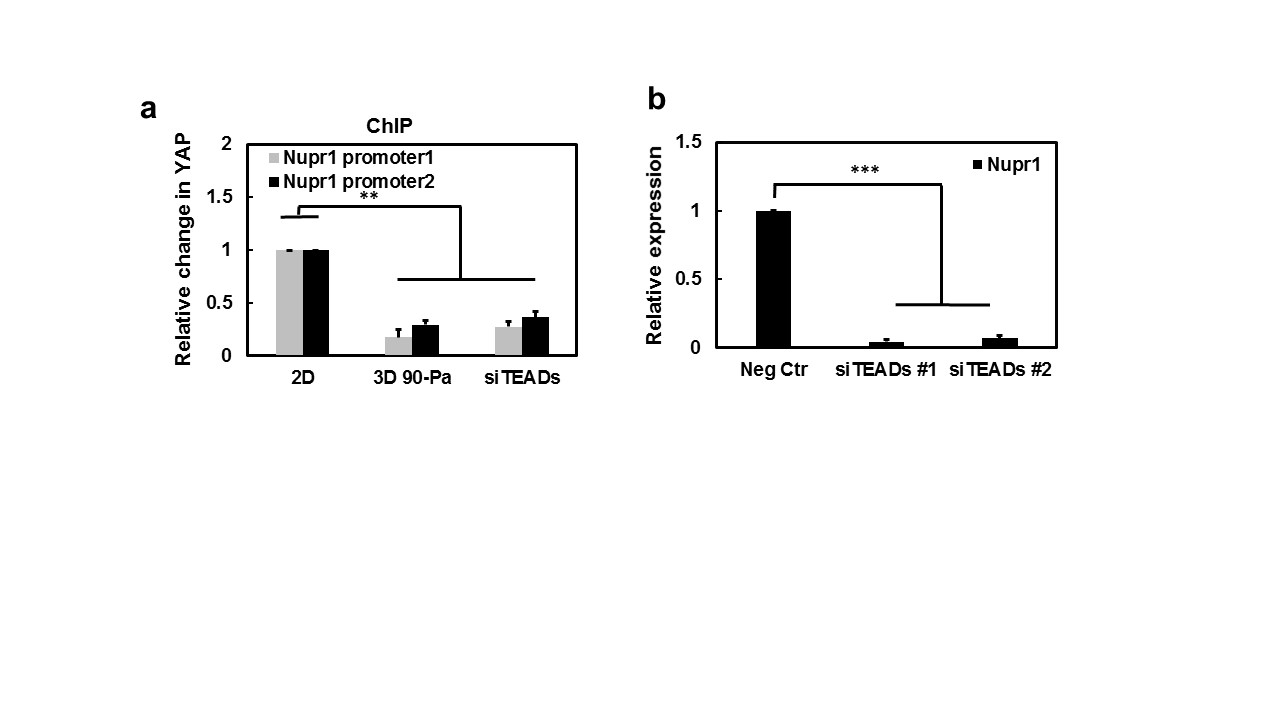
Supplementary Figure 2. Silencing TEADs inhibits YAP binding to *Nupr1* promoter sites and decreases *Nupr1* expression. (a)** YAP enrichments on *Nupr1* promoter region quantified by the ChIP assay. Cells were cultured on 2D rigid dish or in 3D fibrin gels for 5 days, or transfected with TEADs siRNA (including Tead1+Tead2+Tead3+Tead4 siRNAs) #1, #2 for 24 hrs. Cells lysates were collected for ChIP assay analysis. (**b**) Silencing TEADs decreases Nupr1 expression. Cells were transfected with negative control siRNA, or TEADs siRNA #1, #2, for 24 hrs, respectively. Total mRNAs were extracted for quantitative analysis of Nupr1 expression by real time PCR. Mean ± s.e.m.; n=3 independent experiments for all subfigures; ***p*<0.01, ****p*<0.001.

**
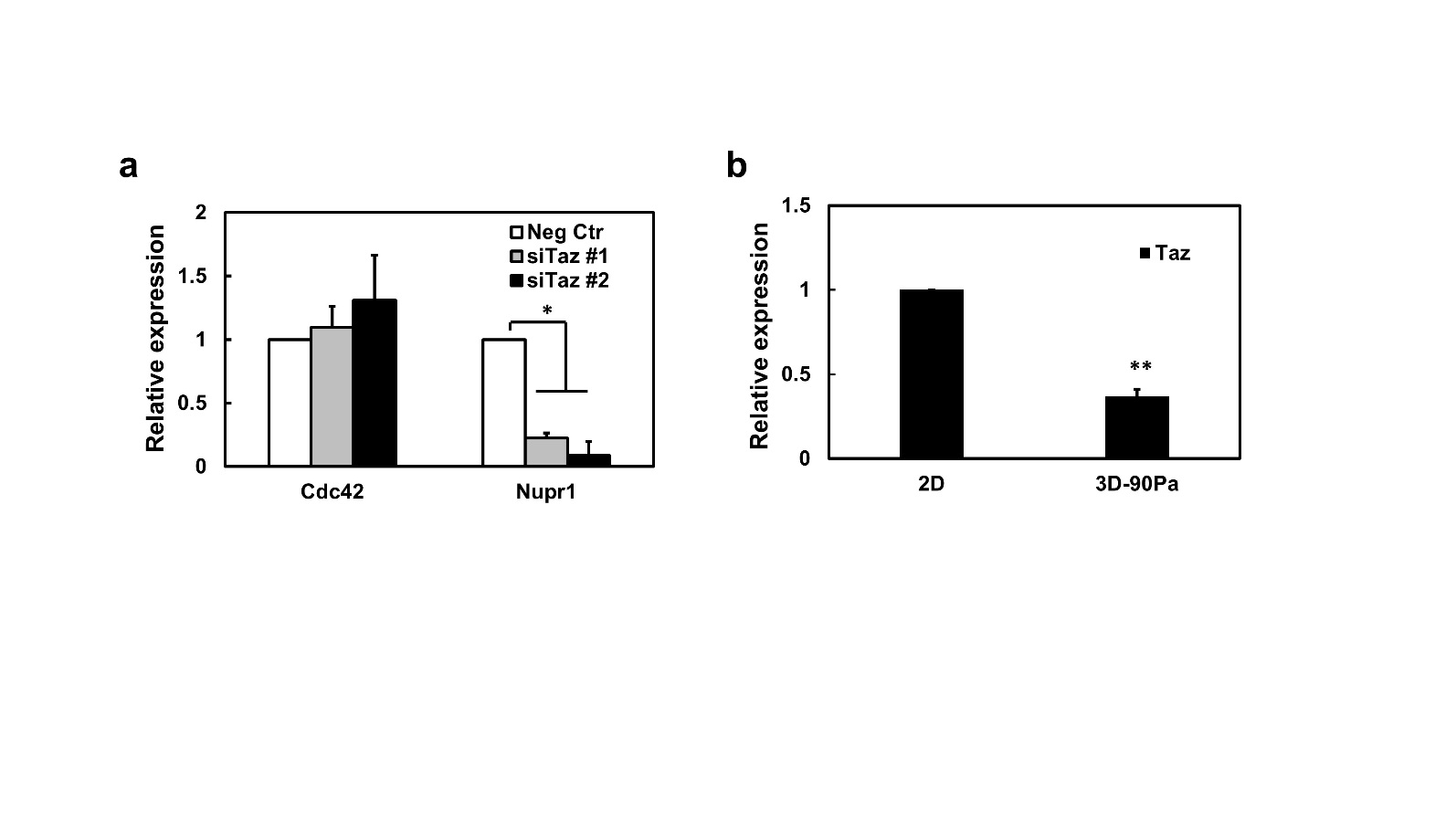
Supplementary Figure 3. Taz expression is low in cells in 3D soft matrix. (a)** Silencing *Taz* decreases the expression of *Nupr1* but has no effects on *Cdc42*. Cells were transfected with negative control siRNA, or Taz siRNA#1, #2, for 24 hrs, respectively. Total mRNAs were extracted for quantitative analysis of *Nupr1* or *Cdc42* expression by real time PCR. **(b)** Taz mRNA expression is lower in 3D fibrin gels than on rigid plastic. **2D:** B16-F1 cells were cultured in plastic. **3D-90Pa:** Control B16-F1 cells were cultured in 90-Pa fibrin gels for 5 days and mRNAs were extracted by real time PCR. Mean ± s.e.m.; n=3 independent experiments for all subfigures; **p*<0.05; ***p*<0.01.

**
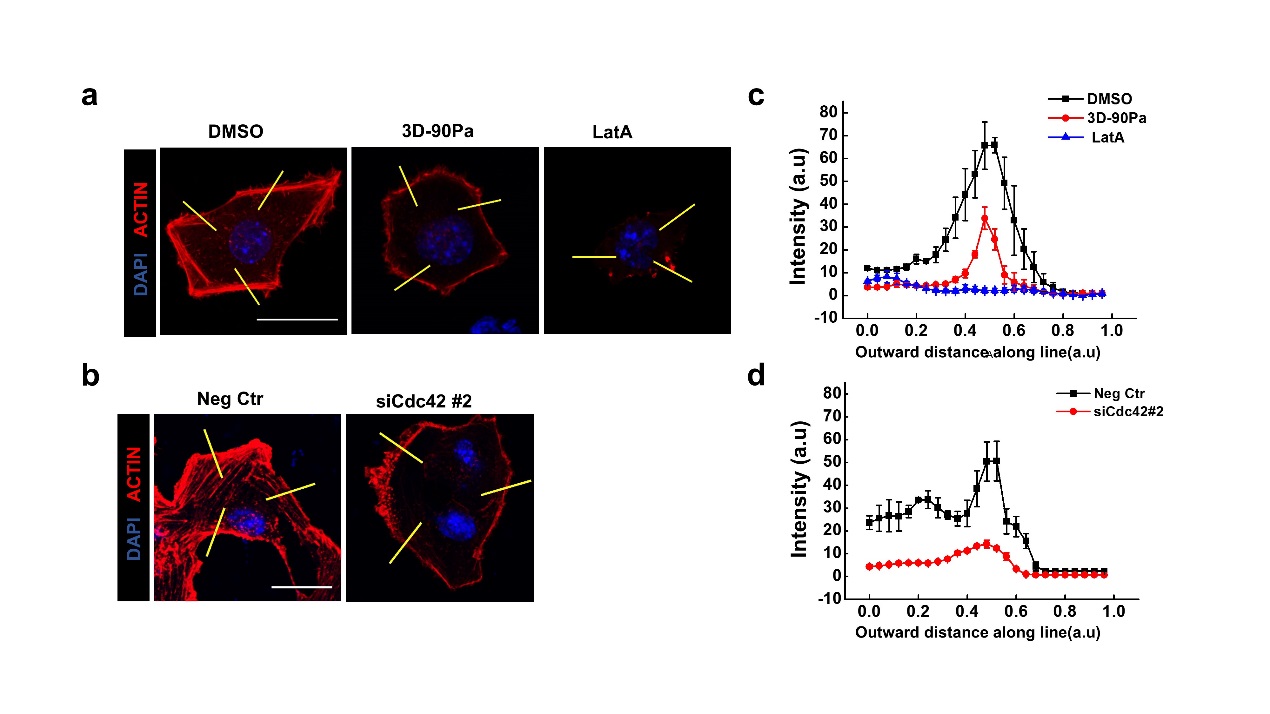
Supplementary Figure 4. Latrunculin A or silencing Cdc42 decreases F-actin.** **(a**) and **(b)**, Cells were stained with rhodamine-phalloidin for F-actin (red) and DAPI for nuclei (blue).Three yellow lines were drawn for quantifying F-actin fluorescent intensity at 3 different cytoplasmic regions. **2D:** cells were cultured on rigid plastic coated with 200ng/ml collagen-1 for 3 hrs. **3D-90Pa:** cells were cultured in 90-Pa fibrin gels for 5 days, then re-plated onto 2D 90-Pa fibrin gels surface for 3 hrs. **LatA**: cells were treated with 1 μM Latrunctulin A for 30 min. **Neg Ctr**: cells were transfected with negative control siRNA for 24 hrs; **siCdc42 #2**: cells were transfected with *Cdc42* siRNA #2 for 24 hrs. Scale bars, 20 μm. **(c)** and **(d)** Quantification analysis of F-actin fluorescent intensities along the 3 lines in each cell.

**
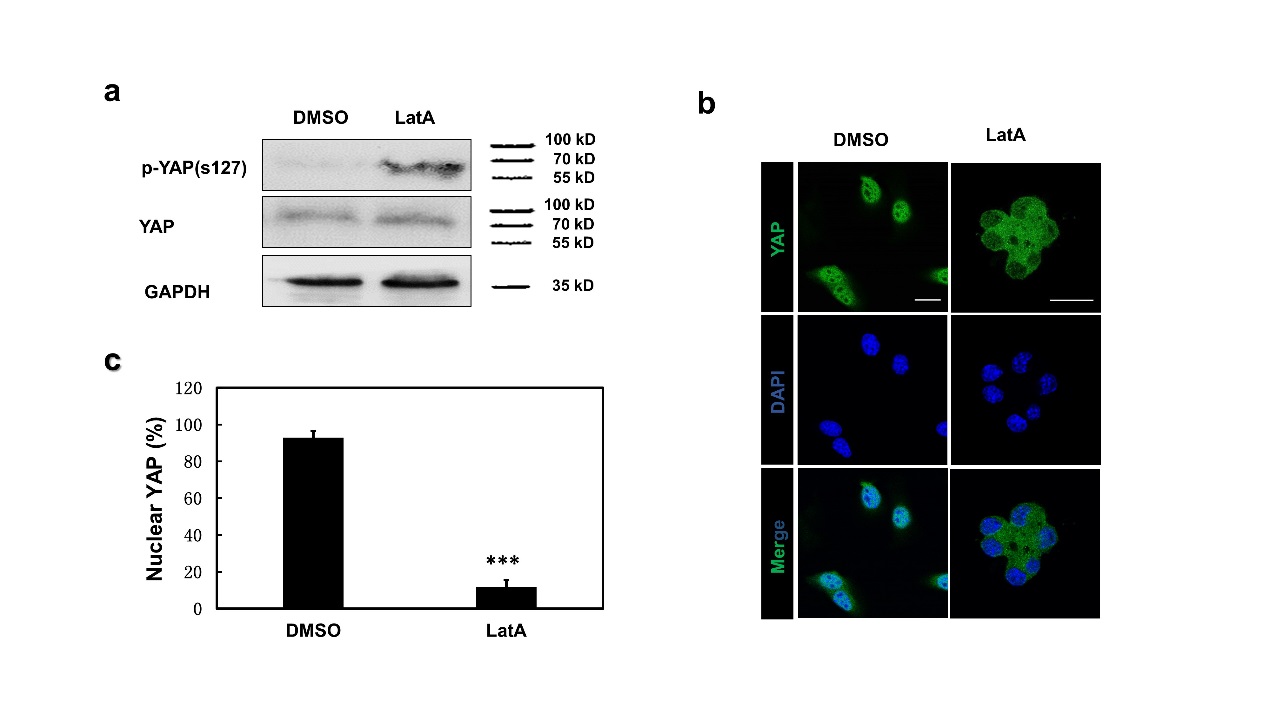
Supplementary Figure 5. Decreasing cytoplasmic F-actin lowers nuclear YAP. (a)** Western blotting for phosphorylated YAP S127 (p-YAP s127) and YAP in whole cell lysates of cells after 12 hrs treatment with DMSO or 1 μM LatrunculinA. **(b)** Confocal immunofluorescence images of YAP and nuclei (DAPI) on 2D rigid plastic (left column) or in 3D 90-Pa (right column) fibrin gel. Cells were treated with DMSO or 1 μM Latrunculin A (LatA) for 30 min. Scar bars, 20 μm. **(c)** Percentage of cells with predominantly nuclear YAP. Mean ± s.e.m.; n=10 randomly chosen view-fields; ****p*<0.001.

**
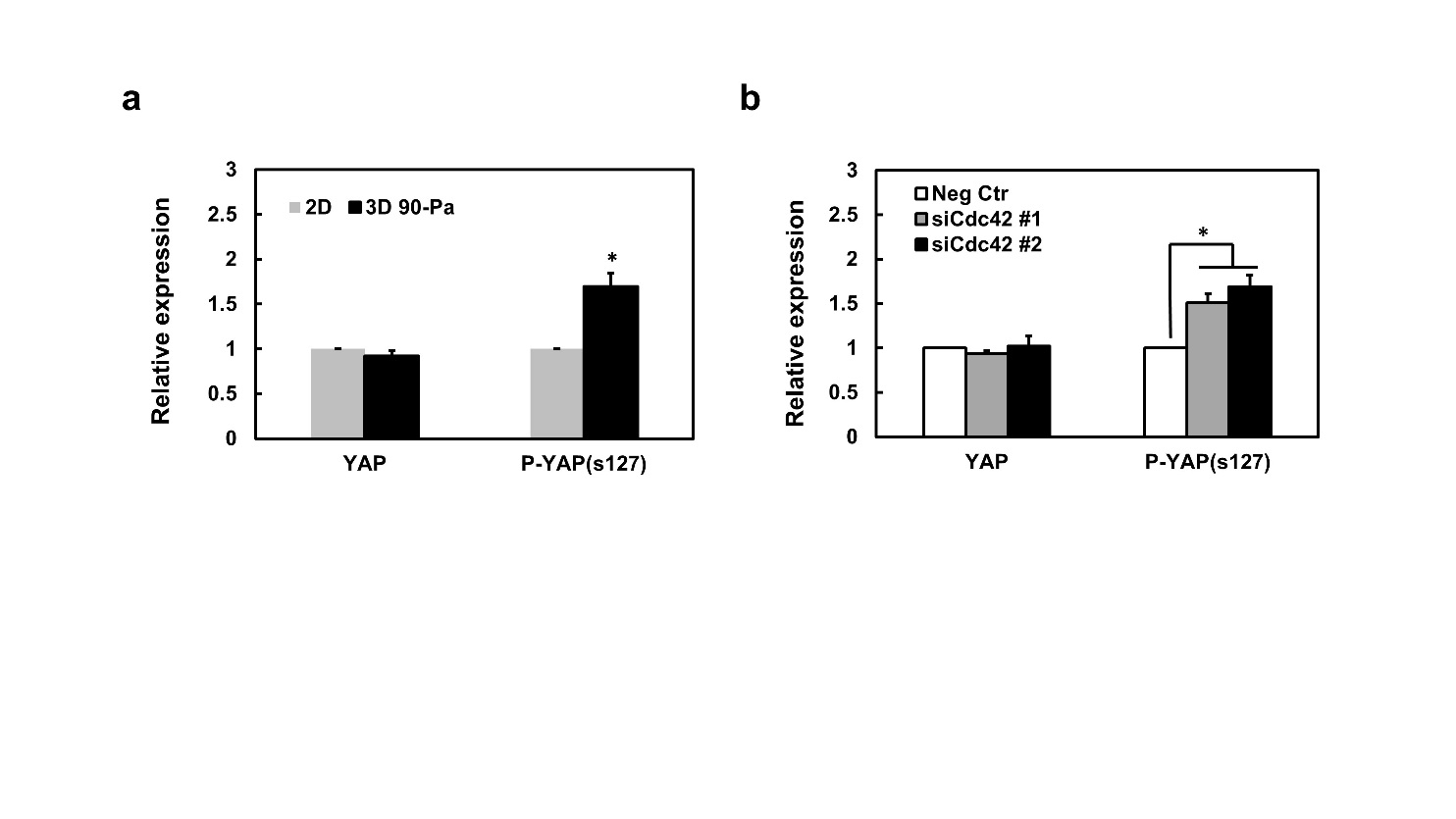
Supplementary Figure 6. Cdc42 regulates phosph YAP. (a)** Protein levels of phosphorylated YAP are higher in 3D fibrin gels than on rigid plastic. **2D:** cells were cultured in rigid plastic. **3D-90Pa:** cells were cultured in 90-Pa fibrin gels for 5 days. **(b)** Silencing *Cdc42* increases protein levels of phosphorylated YAP. Cells were transfected with negative control siRNA, *Cdc42* siRNA#1, or #2 for 24 hrs. Western blots were assayed for quantitative analysis of YAP and phosphorylated YAP. Mean ± s.e.m.; n = 3 independent experiments. **p*<0.05.

**
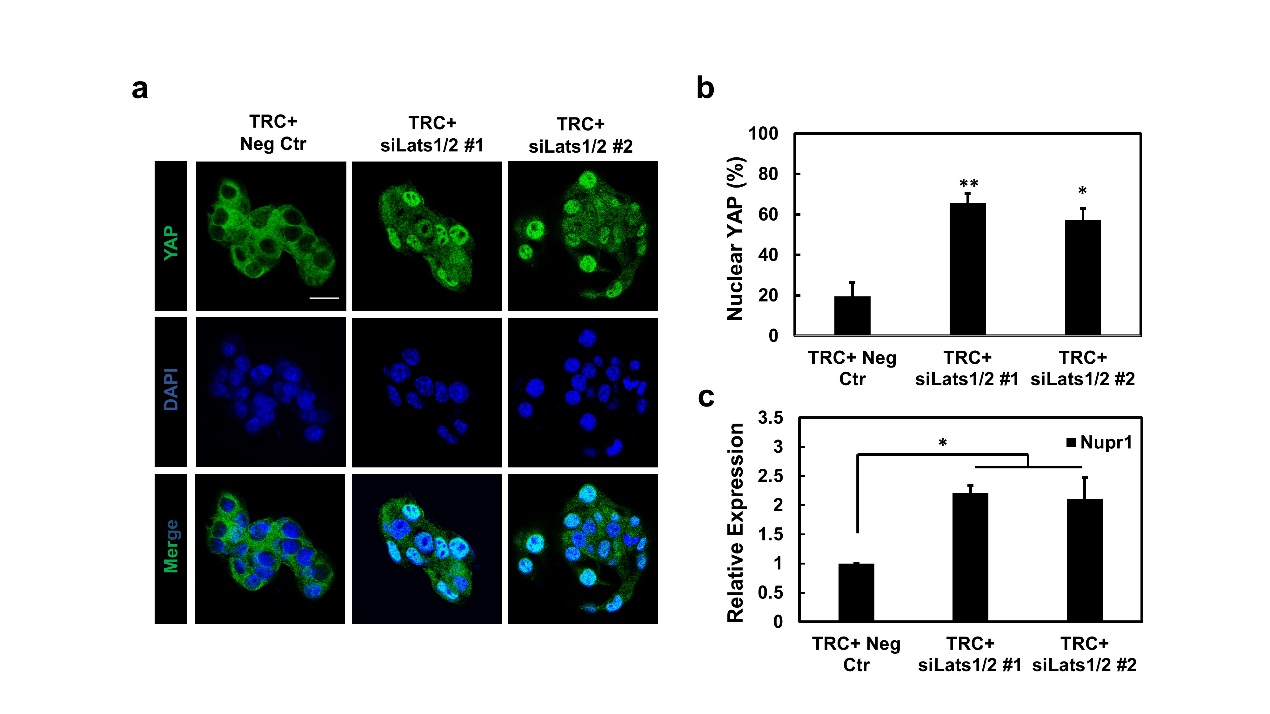
Supplementary Figure 7. Lats1 regulates nuclear YAP and Nupr1.** (**a**) Representative images of immunostaining of YAP (top row), DAPI staining of nuclei (mid row), and merged images of the two (bottom row) of TRCs transfected with negative control siRNA (**TRC+Neg Ctr**, left column), or with siRNA #1 (**TRC+siLats1/2 #1**, middle column) or siRNA #2 to Lats1/2 (**TRC+siLats1/2 #2**, right column) for 24 hrs. Scale bar, 20 μm. (**b**) Quantitation of nuclear YAP under various conditions in (**a**). Mean ± s.e.m; n=10 randomly chosen view-fields from two different experiments; ***p*<0.01; ****p*<0.001. (**c**) Knocking down *Lats1/2* upregulates *Nupr1*. Mean ± s.e.m; n=3; * *p*<0.05.

**
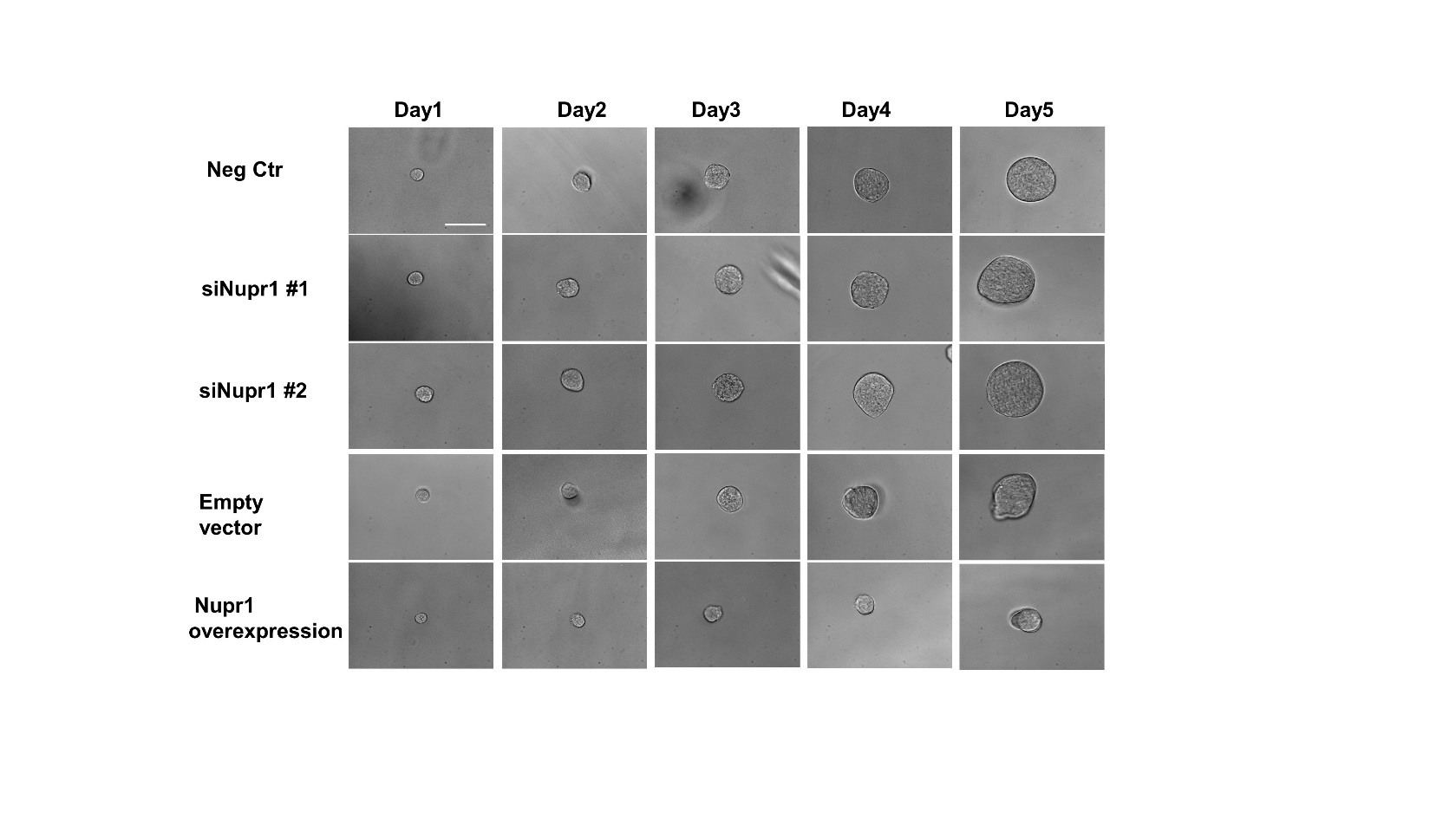
Supplementary Figure 8. Negative regulation of melanoma cell growth by Nupr1.** Representative images of colonies as a function of culturing time in 3D soft fibrin gels after *Nupr1* was silenced or overexpressed. Scale bar, 60 μm.

**
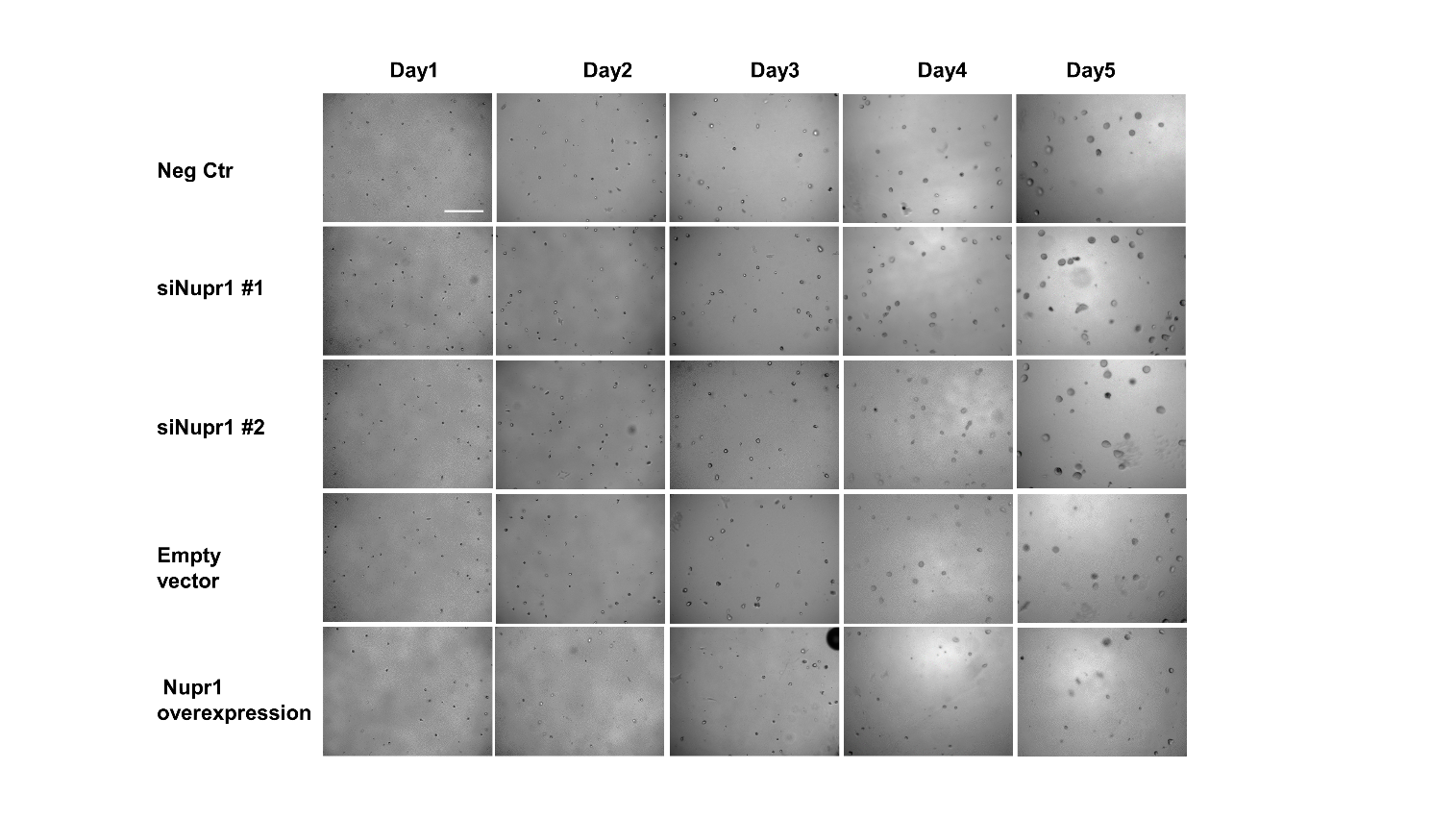
Supplementary Figure 9. Negative regulation of melanoma cell number by Nupr1.** Representative images of colonies as a function of culturing time in 3D soft fibrin gels after *Nupr1* was silenced or overexpressed. Scale bar, 500 μm.

**
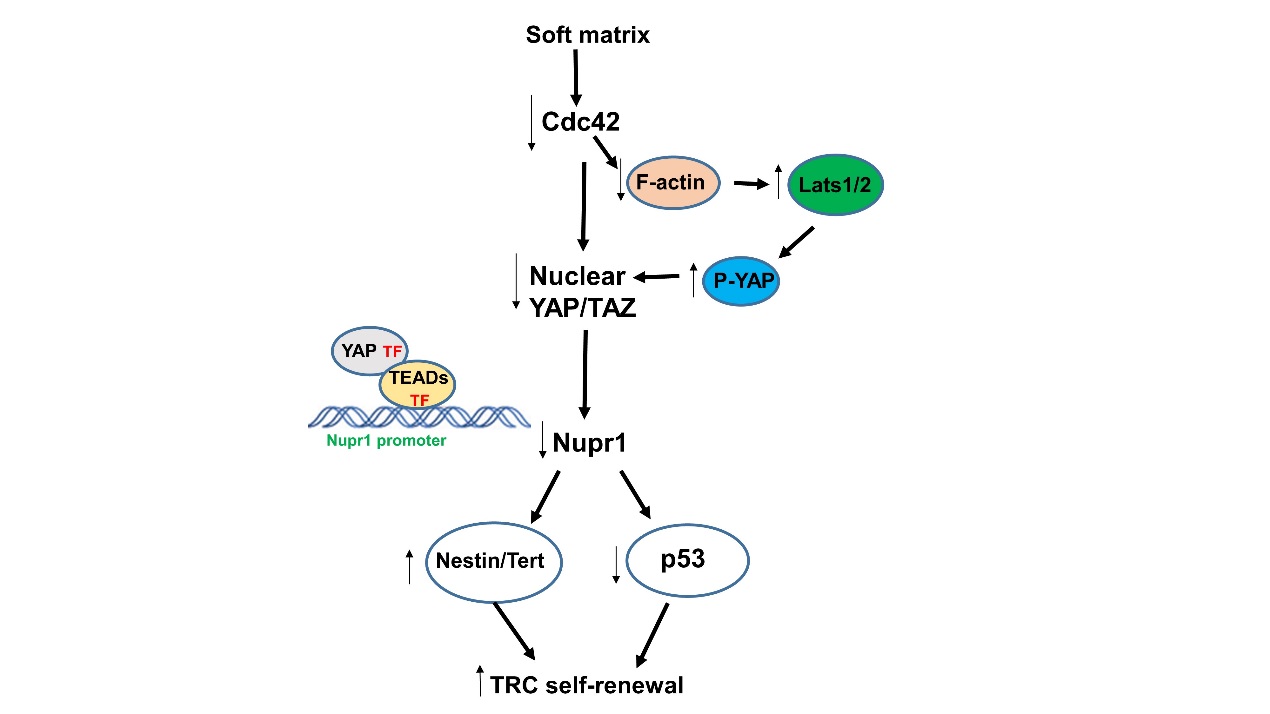
Supplementary Figure 10. A working model for soft matrix mediated Nupr1 downregulation to promote growth of tumor-repopulating cells.**

**
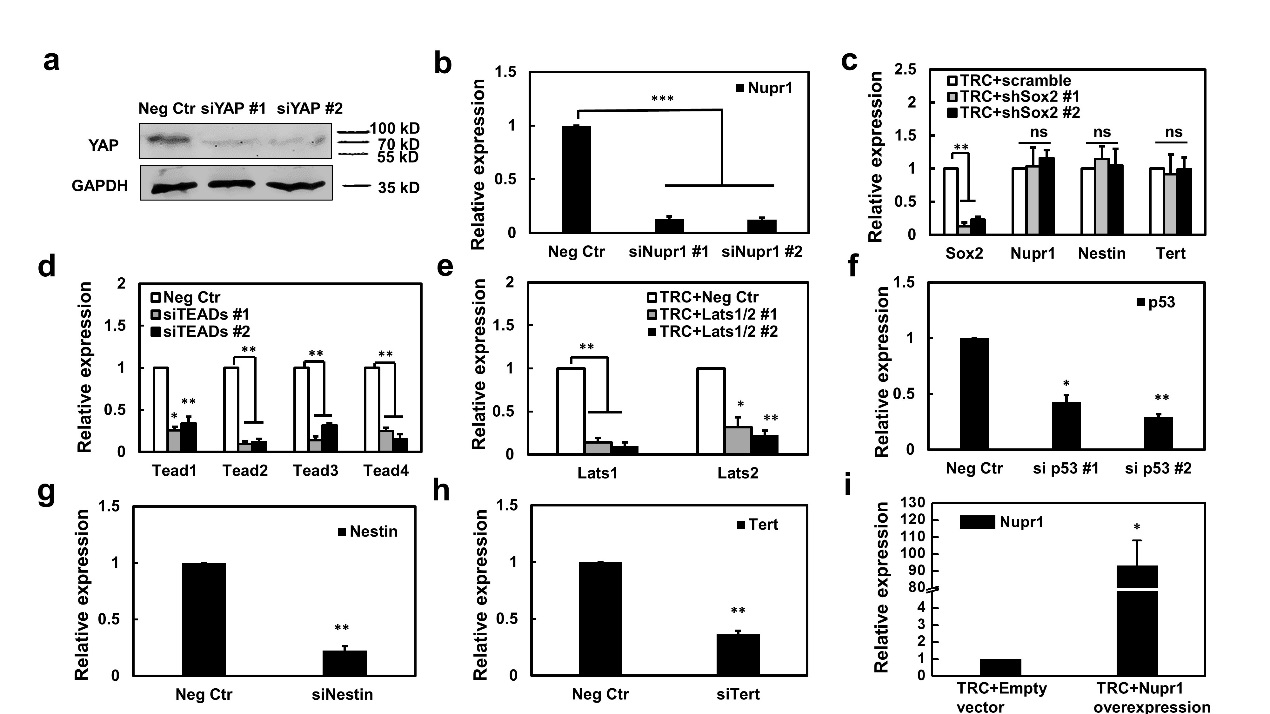
Supplementary Figure 11. Silencing or overexpression efficiency. (a)** Representative images of Western blotting of YAP expression after siRNA knockdown. Cells were transfected with YAP siRNA#1 or #2 and western blot assays were performed to examine the knockdown efficiencies. Three independent experiments showed similar results. **(b-h)** Real time PCRs were performed to confirm the knockdown efficiencies of *Nupr*1, *Sox2,* *TEADs, Last1/2, p53*, *Nestin*, and *Tert* siRNAs. Cells were transfected with negative control siRNA, *Nupr1* siRNA#1, #2, *Sox2* shRNA#1, #2, *TEADs* siRNA#1, #2, *Last1/2* siRNA#1, #2, p53 siRNA#1, #2, *Nestin* siRNA or *Tert* siRNA for 24 hrs. **(i)** Real time PCRs were performed to quantify the overexpression efficiency. Cells were transfected with Empty vector or Nupr1 cDNA plasmid. Total mRNAs were extracted for quantitative analysis by real time PCR. Mean ± s.e.m.; n=3 independent experiments for all subfigures; **p*<0.05; ***p*<0.01; *** *p*<0.001. Note that in (**c**), silencing *Sox2* had no effects on *p53, Nestin*, or *Tert* expression; ns=not statistically significant.

**Supplementary Table 1.** **Sequences of primers for real time qPCR.**

**
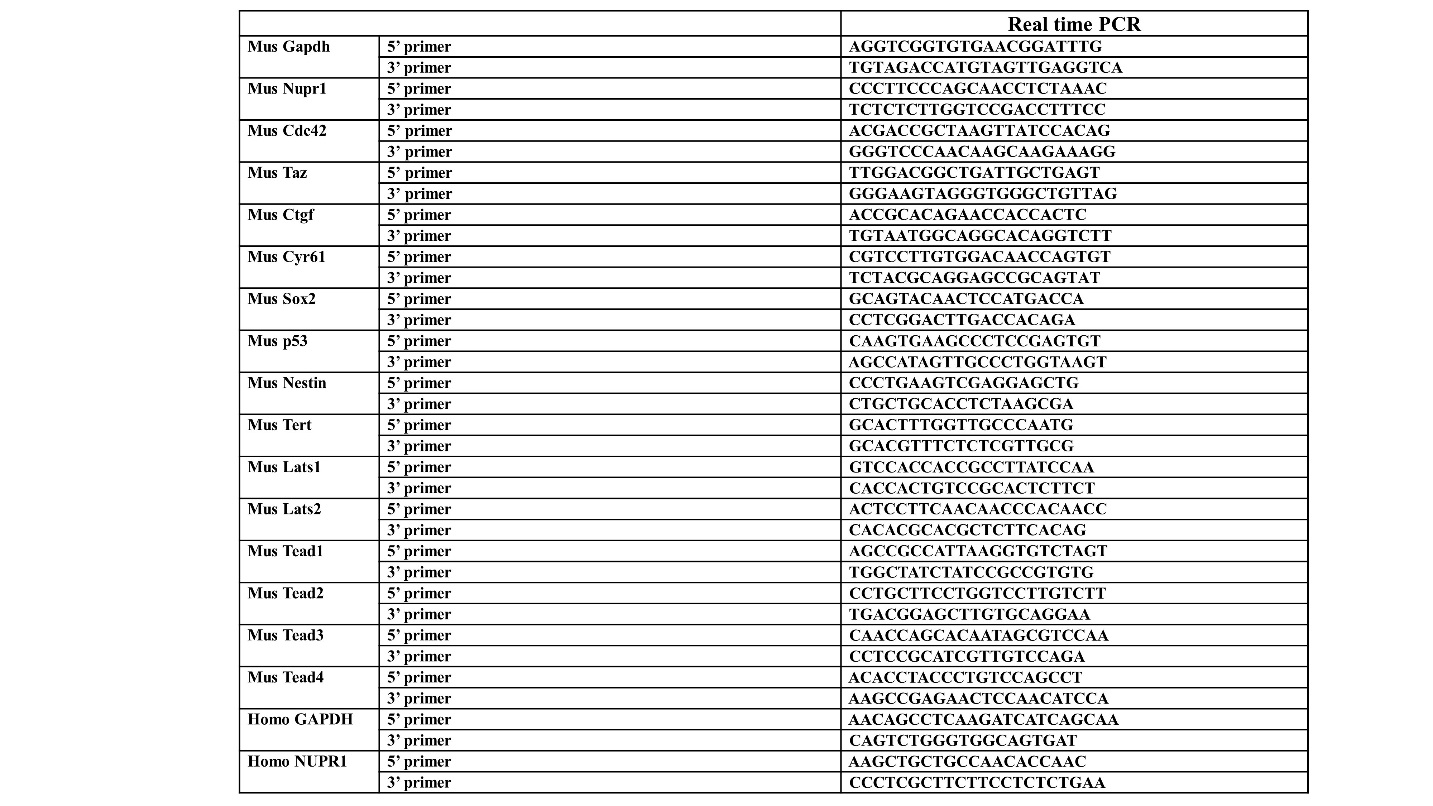
**

**Supplementary Table 2. Sequences of siRNAs or shRNAs.**


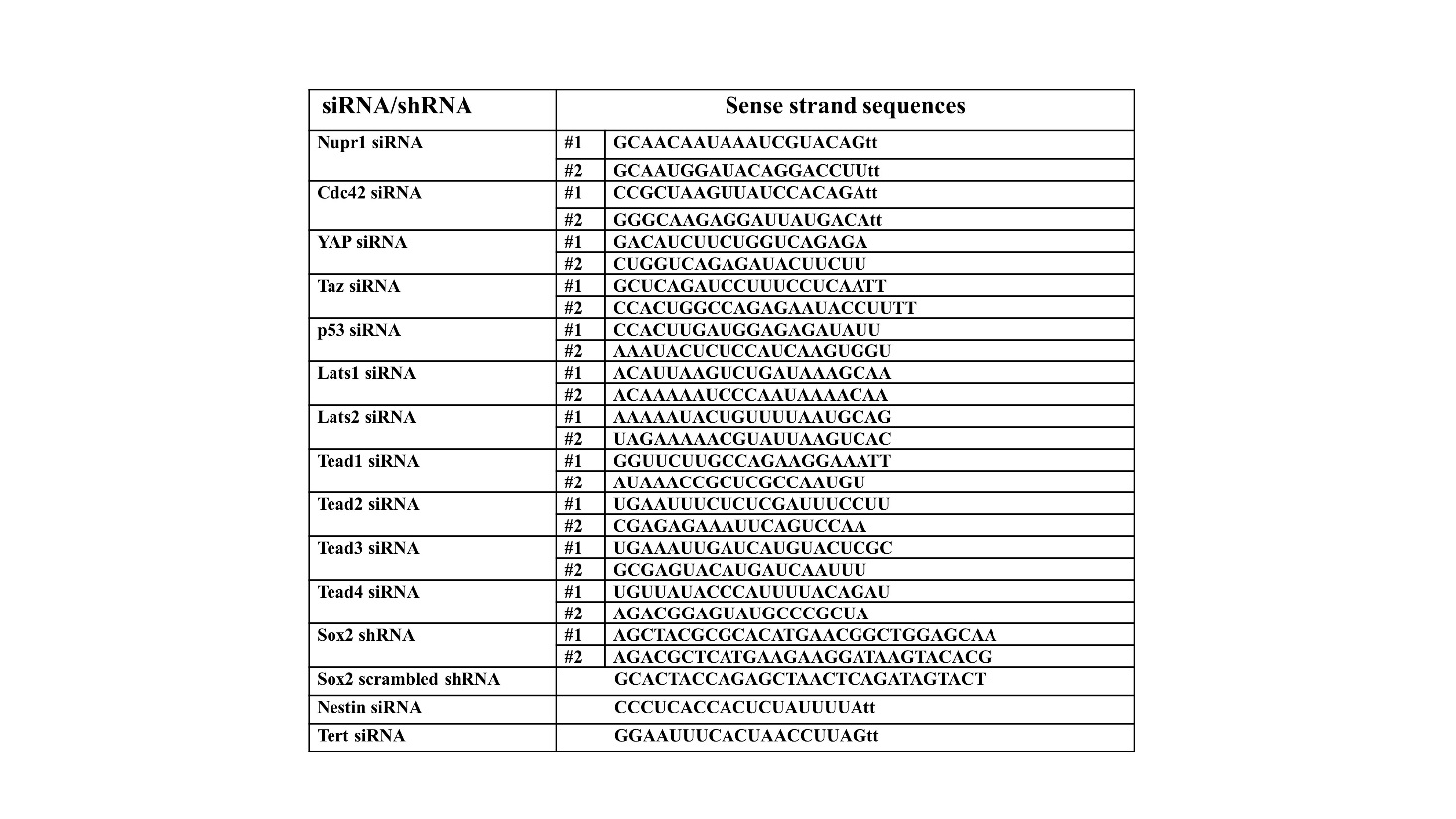

Supplement: Supplementary Information [file oncsis201629x1.docx]
